# Implications of male circumcision for women in Papua New Guinea: a transformational grounded theory study

**DOI:** 10.1186/s12905-017-0406-y

**Published:** 2017-07-27

**Authors:** Michelle Redman-MacLaren, Jane Mills, Rachael Tommbe, David MacLaren, Rick Speare, William J. H. McBride

**Affiliations:** 1 College of Medicine and Dentistry, James Cook University, PO Box 6811, Cairns, Australia; 2 Centre for Indigenous Health Equity Research, School of Health, Medical and Applied Sciences, Cairns, Australia; 3 College of Health, Massey University, Wellington, New Zealand; 4 School of Health Science, Pacific Adventist University, Port Moresby, Papua New Guinea; 5 Tropical Health Solutions, Pty Ltd, Topaz, Australia

**Keywords:** Papua New Guinea, Human immunodeficiency virus (HIV), Male circumcision, Women, Transformational grounded theory, Participatory action research, Decolonizing methodologies, Qualitative research, Pacific

## Abstract

**Background:**

Male circumcision reduces the risk of female-to-male transmission of human immunodeficiency virus (HIV) and is being explored for HIV prevention in Papua New Guinea (PNG). PNG has a concentrated HIV epidemic which is largely heterosexually transmitted. There are a diverse range of male circumcision and penile modification practices across PNG. Exploring the implications of male circumcision for women in PNG is important to inform evidence-based health policy that will result in positive, intended consequences.

**Methods:**

The transformational grounded theory study incorporated participatory action research and decolonizing methodologies. In Phase One, an existing data set from a male circumcision study of 861 male and 519 female participants was theoretically sampled and analyzed for women’s understanding and experience of male circumcision. In Phase Two of the study, primary data were co-generated with 64 women in seven interpretive focus group discussions and 11 semi-structured interviews to develop a theoretical model of the processes used by women to manage the outcomes of male circumcision. In Phase Three participants assisted to refine the developing transformational grounded theory and identify actions required to improve health.

**Results:**

Many women know a lot about male circumcision and penile modification and the consequences for themselves, their families and communities. Their ability to act on this knowledge is determined by numerous social, cultural and economic factors. A transformational grounded theory was developed with connecting categories of: Women Know a Lot, Increasing Knowledge; Increasing Options; and Acting on Choices. Properties and dimensions of each category are represented in the model, along with the intervening condition of Safety. The condition of Safety contextualises the overarching lived realty for women in PNG, enables the inclusion of men in the transformational grounded theory model, and helps to explain relationships between men and women. The theory presents the core category as Power of Choice.

**Conclusions:**

This transformational grounded theory provides a means to explore how women experience male circumcision and penile modification in PNG, including for HIV prevention. Women who have had opportunities for education have a greater range of choices and an increased opportunity to act upon these choices. However, women can only exercise their power of choice in the context of safety. The concept of Peace drawn from the Social Determinants of Health is applied in order to extend the explanatory power of the transformational grounded theory. This study shows that women’s ambivalence about male circumcision is often related to lack of safety, a consequence of gender inequality in PNG.

## Background

Male circumcision reduces the risk of female-to-male transmission of human immunodeficiency virus (HIV) [1–3]. Evidence from three large randomized controlled trials in Africa showed a reduction in HIV acquisition of up to 60% for heterosexual men [1]. Voluntary Medical Male Circumcision is promoted by the World Health Organization as a way of reducing HIV transmission [4]. In Papua New Guinea (PNG), a middle income country in the Pacific, HIV manifests in a concentrated epidemic which is largely heterosexually transmitted. Almost 0.5% of PNG’s population live with HIV [5]. Some populations, such as transgender people in urban areas, have estimated HIV rates of up to 24% [6]. PNG faces many health and development challenges, one of which is how to respond to HIV.

The acceptability and feasibility of male circumcision for HIV prevention in PNG was investigated during two large research studies to inform a national HIV policy response [7, 8]. These studies revealed a diverse range of penile cutting and modification practices and documented a variety of opinions about the implications for women [9, 10]. It is particularly important to understand the implications of male circumcision for women given the low social and educational status of many women in PNG (143 out of 188 countries in the global Gender Inequality Index) [11]. The substantive area of enquiry for this transformational grounded theory study was to explore how women understand, experience and manage the outcomes of male circumcision and penile modification practices in PNG, including for HIV prevention.

The aims of the study were to:

1. Explore women’s understanding and experience of male circumcision and penile modification in PNG;
2. Describe and construct a theoretical model of the processes used by women to manage the outcomes of male circumcision and penile modification in PNG;
3. Identify the implications of results for local-level action, along with national HIV policy and planning in PNG.

## Methods

### The setting

PNG is a diverse middle-income, Pacific Island nation of 6.8 million people, who gained independence from Australia in 1975. Over 800 languages are spoken in this hyper-diverse nation in the tropics, which has great cultural and bio-diversity. The rural majority (85%) live a predominantly subsistence lifestyle. Alongside expanding extraction industries, there is a growing phenomenon of urbanisation with subsequent changes to social and cultural norms, and increasing violence [12]. For women in PNG, life can be challenging. Educational opportunities for many girls and women are limited, with approximately 50% of women in PNG considered literate [13]. While educational opportunities for girls are improving, currently only 12.4% of girls complete secondary school, which is half the number of boys who complete secondary school [14]. Maternal mortality ratio in PNG is the second highest in the Asia-Pacific Region and one of the highest in the world, with an estimated 733 maternal deaths per 100,000 live births ([15]:1223). Of the 111 members of the National Parliament, only three are women [16]. In this study, we worked with women at two sites: a university campus near the national capital; and an oil palm plantation in rural PNG. These sites are culturally, socially and educationally diverse.

### Design of the study

Transformational grounded theory methodology was used for this study. This methodology builds upon grounded theory by incorporating participatory action research strategies and decolonizing methodologies [17]. Consistent with transformational grounded theory methodology, people who participated in the study were considered co-researchers who helped to determine the focus, content and representation of the study findings. In Phase One of the study existing qualitative data from a large, mixed methods multi-site male circumcision study (2010–2012), conducted at four sites (two university campuses, an oil palm plantation and a gold mine) was theoretically sampled for data on women’s understanding and experience of male circumcision. The data consisted of transcripts in English or Tok Pisin (lingua franca of PNG) generated with 861 male and 519 female participants [7]. An analysis of this existing data was conducted by MRM (proficient in Tok Pisin; linguistic quality control was provided by RT) to identify ‘chunks of qualitative data’ that could inform the development of codes and categories about women’s understanding and experience of male circumcision [18]. Codes and developing categories were identified using constant comparison methods, supplemented by memoing [19–22].

In Phase Two of the study, the ‘chunks of qualitative data from Phase One that described women’s understanding and experience of male circumcision were presented to 64 female co-researchers who co-analyzed chunks of data during seven interpretive focus groups. Groups were facilitated by MRM and RT at an urban university and a rural oil palm plantation and were conducted in either Tok Pisin or English (determined by co-researchers at each site) [13 interpretive focus groups]. The chunks of data were discussed by small groups of women in the interpretive focus groups in in a way they themselves determined. “This process evoked a sharing of personal experiences as women discussed their interpretation of the data, their personal positions in relation to the data, and shared much laughter as well” [18]. As a part of responding to, and analyzing the chunks of existing data, the 64 female co-researchers also contributed their unique experiences of male circumcision through guided oral discussions and drawing pictures on ‘storyboards’ [18, 23]. Codes and developing categories were identified using constant comparison methods within each group, based on the oral discussions and pictures. Phase Two also included 11 semi-structured interviews which were conducted to further explore developing categories and ensure theoretical saturation. These 10 women and one man were identified as having specific knowledge about male circumcision, gender and society [20, 24]. Seven of the 10 women had participated in interpretive focus groups, with three women participating in semi-structured interviews only. Oral discussion was recorded and photos were taken of pictures on storyboards. MRM analysed the storyboards, the transcripts, and handwritten notes in the language they were collected in and then used grounded theory data methods of analysis including constant comparison, memoing and initial, intermediate and advanced coding. These processes led to a developing transformational grounded theory [17].

In Phase Three of the study the developing transformational grounded theory, drafted in Phase Two, was presented to six discussion groups during a further round of fieldwork at the two sites (2014). Co-researchers in these discussions were predominantly the same co-researchers who had participated in the original interpretive focus groups. The transformational grounded theory was presented and modified/clarified based on detailed discussions. In addition, the developing theory was presented to and/or discussed with over 100 other relevant stakeholders including health workers, company managers, and employees of non-government organisations. As a result, further modifications were made to the grounded theory that is reported below.

### Sampling and methods of analysis

Phase One: Existing qualitative data from the mixed methods multi-site male circumcision study (2010–2013) was theoretically sampled using grounded theory methods [19, 20]. Data from transcripts of focus groups (M = 36; F = 10) and semi-structured interviews (M = 40; F = 24) were sampled (for the headline results of the of the foundational mixed methods male circumcision study, see MacLaren et al. [7]). Semi-structured interviews (*n* = 9) and focus groups (*n* = 2) were theoretically sampled and analysed from the existing data set. Theoretical sampling was conducted by considering relevance of the existing transcript (purpose-driven), richness of the transcript (layers of meaning within the data), and how the data “maximised opportunities to develop concepts in terms of their properties and dimensions, uncover variations, and identify relationships between concepts” ([25]:143).

Phase Two: Primary data were co-generated with 67 women and one man all from PNG. Co-researchers were theoretically sampled depending upon their characteristics (marital status, gender, from regions where circumcision is usually practiced, from regions where circumcision is not usually practiced and so on). Co-researchers in Phase Two were from two of the four original study sites (a university campus and the oil palm plantation).

Phase Three: a series of six feedback discussions with co-researchers at two sites were facilitated to present the developing transformational grounded theory. During these discussions the grounded theory model was presented, feedback provided by co-researchers about what resonated in the theory and what minor changes needed to be made. In addition, possible local and national level health promoting actions were identified.

Consistent with grounded theory dictum, ‘all is data’ [26], the only published article that had focused on women’s experience of male circumcision in PNG [10], was also included in the dataset as extant data. The process of fracturing, analysing and integrating data was repeated, with codes from the semi-structured interviews, interpretive focus groups and the article by Kelly et al. [10] enfolded into codes already developing out of the initial analysis. Analysis of the data was conducted by the lead researcher (MRM), supported by JM and RT. Using the qualitative software *NVivo*, 162 codes in both English and Tok Pisin were identified from the combined dataset (existing and primary data, including codes identified from the storyboards). These codes were then combined into 93 sub-categories and finally grouped into the core category and the four other categories of the transformational grounded theory.

## Results

Many women know a lot about male circumcision and penile modification. Many women can describe the procedure of circumcision, including the variety of cuts that occur, the local names of the cuts and how they differ from each other. Names women use to describe male circumcision include *raun kat* (round cut) which is the circumferential male circumcision and *stret kat* or *split kat* (straight cut) the longitudinal cut or dorsal slit of the foreskin. These types of cuts have a variety of local names such as *kela (*‘bald-headed’ type of cut)*, kaen blong Sepik* (cut belonging to Sepiks/ round cuts), *V-cut* and *banana split* (straight cuts) [27].

Women know the range of ages that boys and men are circumcised. Women report traditional circumcision conducted on boys from a few days old up until they are young men of 18–20 years of age. The preferred age for circumcision depends upon the cultural setting, the resources of the family who prepares gifts and food to host the feast after the circumcision and where the young man is living. Peer influence can result in circumcision of school boys, university students and older men. Women report male circumcision occurs in a myriad of non-clinical settings, including: *tambu haus* (taboo house)*, haus man/haus boi* (men’s house/boys’ house), by the sea, by the river, under oil palms, in the forest, at school, in dormitories, in school grounds and in the settlements in urban areas. Some women report circumcision occurs in clinical settings such as clinics or a hospital, but this is much less common. A female university student said,

“*They boiled the razor blade, you know the razor blade, they boiled the razor blade and then they removed it and then they did a slit, but we were not allowed to know about it. We knew about it but were not allowed to go near the boys yeah, the boys stayed in the room and we stayed away from them (laughter)”* (Pacific Adventist University (PAU) Semi-structured interview (SSI)).

**Fig. 1:**
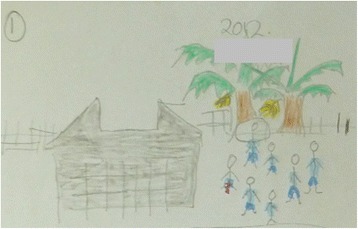
The transformational grounded theory model

It is not uncommon for primary school and high school boys to cut each other’s foreskins. This can result in serious injury and family disharmony. A group of women drew a picture on a storyboard that illustrated this type of informal cutting amongst students (Fig. 1).

Women know about male circumcision and penile modification from a variety of sources. They know because they are sexual partners, participants in community feasts, through observation, health practitioners and family members. Most women find out if their male partner has been circumcised once they are in a sexual relationship with them. One woman explained, *“Only their wives will find out or whoever is having sexual relationship with that man”* (Divine Word University (DWU) SSI). Another co-researcher said, *“Basically the girl will notice during, like when they having sex and then the boy tells the girl”* (PAU SSI). Women who are nurses or health workers do not usually perform the circumcision, but they are often asked to provide bandages, gauze and medications and assist boys and men. In addition, co-researchers reported sharing sanitary napkins to assist boys in the management of bleeding post penile cutting in a school dormitory.

Some women know about male circumcision because they are family members. When a man has been circumcised, women are often asked to prepare less food or different types of food such as dry *kaukau* (sweet potato) so that the young men do not have too much fluid, thus reducing the need to urinate. *“Ol bai stopim kaikai, stopim wara ol disp’la kain”* [They will stop eating, stop drinking water or things like that] (Oil Palm Plantation (OPP) SSI). The preparation of special food often falls on the mother.

Some women are happy for men to be circumcised, especially if they come from traditionally circumcising areas. They see circumcised men as manly, capable of caring for a family, strong and healthy. They are able to take their rightful place in the community. Others think circumcision is healthy or consistent with religious teachings. However, if circumcision is not a traditional cultural practice, women think men are motivated to be circumcised for different reasons. Increased attractiveness to women is frequently cited as the main reason men from non-traditionally circumcising areas get circumcised. *“Taim ol katim…em isi lo kisim ol liklik yung gels tu, kain olsem”* [when they get circumcised/cut… it is easy to attract young girls for intimate or sexual relationships, something like that] (OPP Interpretive Focus Group (IFG)).

*“So why do boys do the circumcision? Basically to attract the women, in a sexual way…the trend is that boys have a lot of girlfriends. If you have a lot of girlfriends ‘yu man’* (you are a man)*…so they circumcise themselves and the more women they sleep with, so it kind of, they also taken on the health side, but psychologically they want to be want seen as tru man* (real man)*”* (PAU SSI).

Women have mixed views of male circumcision but unanimously oppose penile modifications, such as inserting objects or injecting fluids. Women describe men inserting ball bearings under the skin of their penis (the number of ball bearings reported ranged from 2 and 15), and inserting parts of toothbrush handles and other objects in the wall of their penis. One co-researcher from the oil palm plantation explained her response to these practices,

*“So mi bin go againstim displa, lo olsem katim stret na lo sait blo health or displa kain safety em orite tasol, lo somapim bearing na wanem kain ol samting em ino gutpla”* [I am against these practices, if it is like a straight cut for health reasons or this kind of safety, that is alright, sewing in bearing or something like that is bad] (OPP SSI).

Some women describe men injecting fluids into their penis. These women knew details about how long the substance stays in the wall of the penis, how hard the penis becomes and the type of damage this causes women when men have sex with them. Some co-researchers had assisted women in their village to seek medical help due to the excessive bleeding that had occurred after sex with a man who had injected fluid into his penis. Women know a lot about male circumcision and penile modification, despite it being a culturally taboo subject for them to discuss in public or with men. Women are often asked to take active, supporting roles when men are circumcised.

Women know about the consequences of male circumcision or penile modifications for their relationships, including a potential change in the nature of their sexual relationship, or a perceived risk that men will increase the number of sexual partners they have. The level of detailed knowledge women have about male circumcision and penile modification raises questions about the nature of gendered, taboo knowledge. Thus a property of this category is the taboo nature of male circumcision. For women who come from traditionally circumcising areas, their fathers, mothers or other family members explain that circumcision is a taboo topic. *“Cultural reason yeah… if I’m openly talking with my partner (about male circumcision) then I’m disregarding culture, because they don’t talk about it”* (DWU SSI). Co-researchers reported negotiating with their husbands, brothers and uncles to speak to researchers about male circumcision, with one saying,

“*When I asked them (the brothers), I said I’m going for an interview with this lady, so they felt sorry for me and they said “oh, it’s okay” for me to tell you.” (PAU SSI).*

For some women, just keeping safe is their focus and they keep quiet about what they know. There are potentially negative consequences, perhaps even to their own safety, if they speak about this men’s business to the ‘wrong’ people.

**Fig. 2:**
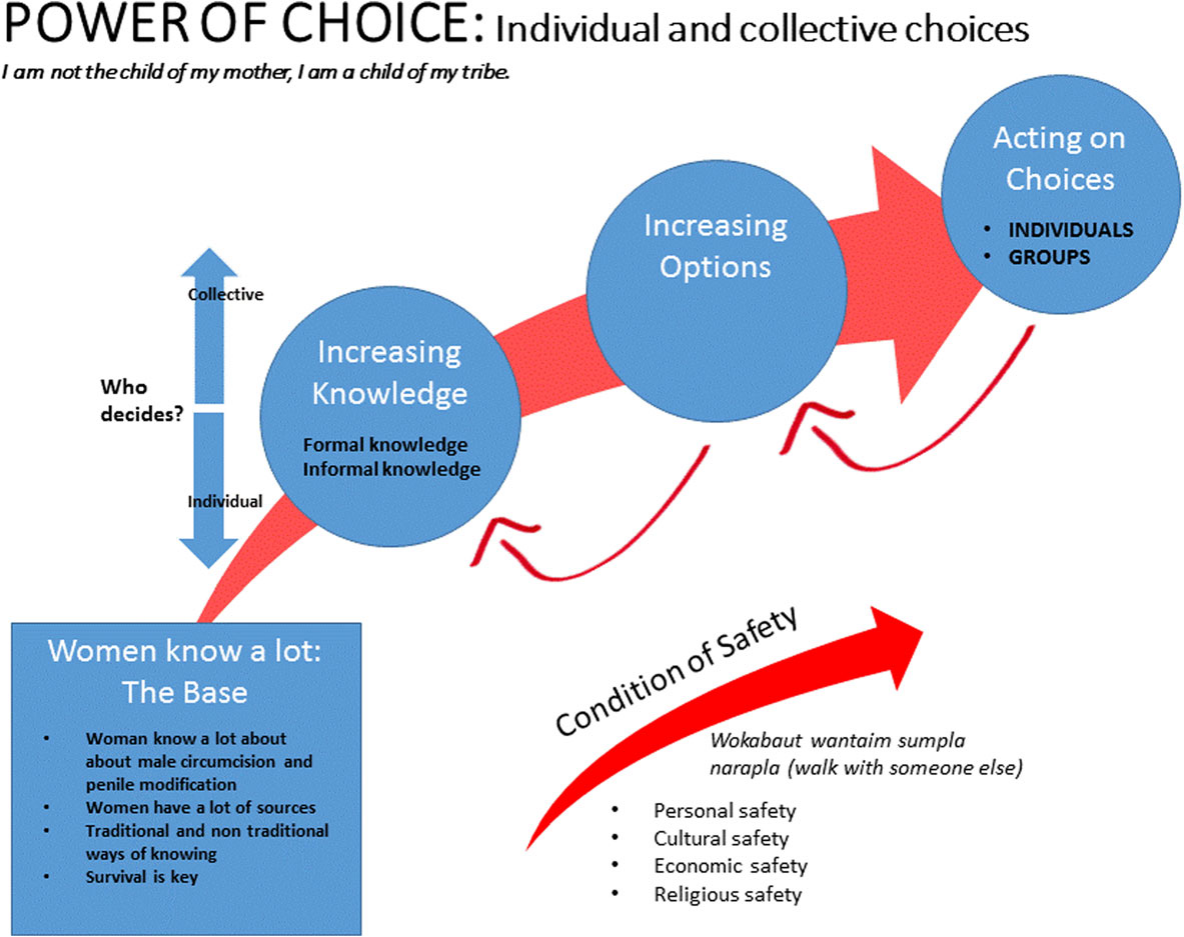
Storyboard picture of school boys cutting each other’s foreskins under banana trees at the back of a school building (name of school covered)

**It is from this data that the First Category of the grounded theory is: Women Know a Lot**. This is the Base category (Fig. 2) and represents the plethora of knowledge women have about male circumcision and penile modification. Despite these activities being seen as ‘men’s business’, many women know when, where, how, by whom, costs, medications required and many other details about male circumcision and penile modification. Women know because they are wives, girlfriends, sisters, mothers, health workers and/or teachers. Although women know a lot, they are careful with this knowledge. Speaking about male circumcision and/or penile modification outside of accepted social or gendered groups can result in a threat to women’s safety.

From this Base category, we now explore how an increasing knowledge can influence women’s experience of male circumcision and penile modifications.

**The second category of this transformational grounded theory is Increasing Knowledge.** Women report that if they have more knowledge such as a formal education (high school, tertiary) or informal training (health worker training, community level training) they have greater status in their community. This change is almost always positive for the woman and her family. It affords women more options and greater autonomy. She is more likely to be a person in the community that women (and some men) go to if they have a problem. An educated woman has opportunity to move beyond usual social and cultural norms and mores. When discussing male circumcision in an interpretive focus group, one woman with a qualification in the agricultural field was referred to as the woman who had a *pepa* - a qualification. This woman was deferred to by other women during the group. Deferring to a leader known in PNG as a *big man* or *big meri* (literally, important man or important woman) is the norm. PNG is comprised of hundreds of collective societies, each based upon values of group benefit. Important as a research finding is how formal education or training is highly valued by community members, and with this valuing comes possibilities not available to women who have not had educational opportunities. Educated women in PNG are invited to be experts in not only their field of expertise, but also in many other aspects of community life.

Women have to be committed to undertaking education or training as individuals, but they also require the support of others in their family and broader community. A dimension of this category is the collective support required for a woman to participate in education or training. Participation in education or training requires both individual commitment and the approval and support by men and other women in the community. This includes approval by a woman’s husband if she is married, or approval by a woman’s father or brothers if the woman is not married. When a woman participates in training, other women will take on some of the responsibilities of the trainee, and thus there is a community-level impact. Women who train as volunteers, for example for *Tingim Laip* (a community-based non-government organisation focussed on preventing HIV), must assess the benefits and risks of participating in informal education. One of the benefits of women participating in education and training is the increased number of options that result. Specifically, increasing knowledge results in a change in the way women understand and accept male circumcision. One co-researcher explained,

*“…mi no bin hamamas bipo taim mi bin lainim dispela samting…but bihain gen mi bin go insait long wok blong Tingim Laip, ol tok ‘dispela em safe, dispela em safe na ol workim em safe so yupela no ken kross*.” [I was not happy before I learnt about this (male circumcision for HIV prevention)…but after I began the work with *Tingim Laip*, they told us ‘this is safe, this is safe, they can do this safely so don’t get angry’] (OPP SSI).

Women who have a formal education or some training opportunities have greater status in their community. This increased status brings a greater number of options for managing the implications of male circumcision and penile modifications, and also increases the range of choices in other areas of a woman’s life.

**The third category of this transformational grounded theory is Increasing Options**. If a woman has an education or some form of training her status in the family and community is increased and she will have more options. These options will be about whom she can have relationships with, the nature of those relationships, her power in those relationships and who she can influence. A woman with an education will have more choice about with whom she has intimate relationships, whether a man’s circumcision status will influence that decision and whether to make a decision about male circumcision for her sons. As one educated co-researcher explained, “*We can say if we want*” (PAU SSI). The increasing options available to trained and educated women lead to feelings of confidence and a plan to act. *“I wasn’t aware of that, now that I’m aware, believe me, I’m going to have all my boys in my family, I will do it and I’m gonna do it. I will have to get them circumcised, that’s it.”* (DWU SSI). Increasing options become available to women with increased knowledge and an enabling environment. A co-researcher was asked if women have influence in their families to give information and help them make healthy choices. The co-researcher responded, “S*ome would, some might not, depending on different factors. Maybe their education levels, back to the cultural, how men see women in their society and stuff”* (PAU SSI). However, it does not always follow that once a woman knows about her options that she can act.

**The fourth category of the transformational grounded theory is Acting on Choices**. The women who have increased knowledge, increased status and an increased range of choices can act on their options. They can act to keep themselves and their families safe, choose healthy sexual relationships, choose the best for their male children and discuss and agree upon options with their male partner. Some women make decisions that are not culturally sanctioned or are against family wishes, while some women convince their husbands to act for themselves, for their young or teenage sons to be circumcised. Only a few women take this type of action. For women to act, they need to be convinced about the benefits of the action, have power to make a decision, have resources if the decision brings negative consequences (for example, familial proximity to other powerful men, or a personal income) and/or have the support of family. If a woman is educated, male circumcision for her husband or children is more likely to be a joint decision between her and her partner.

*“They (women) can do things to satisfy their boyfriends and husbands, like when they are, when they become their husbands wife, they need to satisfy all of the needs that the guy has so instance of sexuality they can also satisfy the need, then the guy to respect the woman, the woman also has rights to when to have sex and when not to have sex”* (PAU SSI).

Women describe a dimension of risk associated with moving beyond social and culturally sanctioned roles, particularly when it comes to sexual health issues, such as male circumcision. If someone takes offense at a woman’s involvement in ‘men’s business’, whether that be her partner, her sons or other family or community members, there can be serious physical, social, cultural and economic costs for the woman and her family/community. Compensation can be requested of the woman’s clan and she can experience isolation and a reduced status in the community.

Women without the increased status afforded by an education are most likely to leave the decision about male circumcision to their male partners and son/s, even if they think male circumcision is a good thing. Women report not wanting to face negative consequences when their son/s grow up. Some women fear their son/s may ‘blame’ them for being circumcised. If a circumcised male were to grow up and object to the decision of his mother to have him circumcised, there could be serious social and cultural consequences as adult children in PNG are responsible for providing every aspect of care for aging parents. An older woman said she would not want to have her son circumcised in case he was unhappy and therefore not care for her later in life (OPP (IFG)). One woman explained, *"An infant should not be* (circumcised)*…it should be done to those big enough to understand what's going on with them so they themselves should decide what's done with them"* (DWU Focus Group Discussion (FGD)). Women who take action to have their sons circumcised, counter to cultural and social norms, usually rely on their knowledge about health and HIV transmission to make the decision. There is both an individual and collective dimension to this category.

Women in PNG experience extreme rates of family and sexual violence [28] and this influences many day-to-day decisions a women makes - where she walks, how she speaks to her partner or family members, to whom she says yes and no. Women often walk together to ensure safety. As one co-researcher described, *“Walkabout wantaim sumpla narapla, tasol walkabout wanwan em i no sef”* (walk with someone or others but walking alone is not safe) (OPP SSI). If a woman does not act according to societal norms, her personal, cultural and/or economic safety can be at risk. Such behaviour can also affect her connection to her religious community, if she acts in a way that is not ‘acceptable’. A woman’s future ability to act on choices may be threatened by her partner, his family members of her partner or members of the village/community. The seeming trajectory of the transformational grounded theory model, as represented by the large red arrow (Fig. 2), does not always go in one direction. If a woman’s safety is compromised, she may return to the place she knows she has increased options but not be able to act, as represented by the red arrow between Acting on Choices and Increasing Options. A woman with knowledge may also know she has increased options but may feel she needs more education or training to increase her legitimacy to act, as represented by the red arrow moving between the categories Increasing Options and Increasing Knowledge.

**The ‘core’ category of this transformational grounded theory is Power of Choice.** *Power of Choice* is the overarching category that represents the central phenomenon present in each of the other categories [19]. As the overarching category it brings together concepts which explains how women understand, experience and manage male circumcision and penile modification in PNG. Power of Choice includes dimensions of both individual and collective power. A person who has the ability or freedom to direct or influence outcomes for themselves and others in the context of family or clan has power. The core category of “Power of Choice” for this transformational grounded theory, is an in vivo code (code using participant’s actual words) gifted during a semi-structured interview with a young female student at PAU (PAU SSI). This core category has two key concepts, Power and Choice, that when combined, creates a phenomenon that encompasses the four categories and intervening condition of the theory. Choice is both an individual and collective experience, reflecting the cultural context in which the women in PNG live. The concepts of Power and Choice contextualise women’s reported experience of male circumcision and penile modification. Women with more education have more power of choice. Power enables an ability to influence and make decisions. Women require both knowledge and an enabling environment to have the power to make and act upon a decision. Choice refers to a woman’s power to choose between the possibilities available without fearing negative consequences.

A key dimension of the core category is the collective nature of cultural and social organization in PNG. The experience of collectivism is encapsulated in the phrase, *“I am not the child of my mother, I am a child of my tribe”* (PAU SSI). This predominantly collective society is organised around a context specific, reciprocal *wantok* (literally ‘one-talk’) system. The majority of the over 800 language groups in PNG are patriarchal, which defines the nature of relationships between women and men, women and their male children and between women. This context influences how individual women can or cannot make decisions and how women may experience and/or influence the decisions of men or other women. Western assumptions about the nature of a mother-child relationship, with related assumptions about women being able to decide about the circumcision of their male children rarely hold in PNG.

## Discussion

This transformational grounded theory explains how co-researchers understand, experience and manage male circumcision and penile modification in PNG. The theoretical model centralises the core category, Power of Choice, while connecting categories of Women Know a Lot, Increasing Knowledge, Increasing Options and Acting on Choices (Fig. 2). Properties and dimensions of each category are represented in the model and anchored in the text, with evidence from co-researchers. Strauss and Corbin describe intervening conditions as broad conditions that bear upon action [25]. Safety of women is an intervening condition that affects all categories in this transformational grounded theory. Safety contextualizes the overarching lived reality for women in PNG, invites the inclusion of men in the transformational grounded theory model, and explains relationships between men and women in this study.

Encompassed by the core category of Power of Choice, the four categories of this transformational grounded theory explain how women understand, experience and manage the outcomes of male circumcision and penile modification in PNG. We locate this grounded theory in international health literature, by identifying ‘social determinants of health’ [29] as the theoretical code that increases the explanatory power of the grounded theory. Glaser explains theoretical codes conceptualise how codes relate to each other when integrated into a theory. Theoretical codes are also emergent “they weave the fractured story back together again” ([30]:72).

In this grounded theory, the theoretical code of ‘social determinants of health’ is applied in order to contextualise the transformational grounded theory, which is focused on local understandings of male circumcision to inform health promoting action, including HIV prevention. As a theory of public health, social determinants of health provides a structural explanation as to why people experience different health status when living in the same communities [29]. The ten social determinants of health identified by Wilkinson and Marmot are: the social gradient; stress; early life; social exclusion; work; unemployment; social support; addiction, food and transport [29]. As shown in the reporting of this grounded theory, many women in PNG endure negative aspects of these social determinants of health. While many women know about male circumcision (Category 1), including for HIV prevention, there are impediments such as a lack of social status in a predominantly patricidal society. There is also individual and community-level stress due to continued exposure to violence [31–33], and in a collective society, potential social exclusion for taking individual health promoting actions outside of social, cultural and religious norms (for example, supporting the circumcision of their young sons when their church forbids it) [10].

These impediments also include a lack of opportunity to participate in formal education (Category 2), a reduced ability to explore options when compared to their male counterparts (Category 2) and reduced opportunities to act upon health promoting decisions (Category 4), with a sense of safety (Intervening condition). This sense of safety is critical to health – with peace referred to as a fundamental pre-requisite for health in the Ottawa Charter [31]. A social determinants frame assists us to weave greater explanatory power into this grounded theory. It also has the potential to inform and transform local level action, holds possibilities for national health policy action in PNG and suggests lessons for international action for HIV prevention. As Dean et al. explains, effective action on the social determinants of health:

*“requires having sufficient knowledge of the mechanisms influencing health inequities and adopting a conceptual framework that not only clarifies the relationship between social determinants and health inequities, but also helps to identify entry points for intervention”* (34:1).

Results from this study will inform policy makers of the potential risk of negative, unintended consequences for women that may result from proposed health prevention programs, such as mass male adult circumcision programs.

### Movement towards action

Transformational grounded theory draws upon participatory action research approaches and includes an action component that is conducted in a partnered and power sharing manner [17]. Although the study concluded, health promoting action continued. As a result of recommendations from women during the study, brief intervention sexual health training was facilitated for supervisors (*n* = 22) and employees (*n* = 327) of the oil palm plantation by a senior sexual health practitioner [34]. Health workers (*n* = 22) working with the oil palm company, along with clinicians from provincial health services, were also provided with a two-day course to discuss implications of male circumcision, penile modification, sexual and relationship issues [34]. In addition, three other research projects were planned with funding sought to enable the health promoting work. This research continues to create opportunities for change at the local level. The strength of this grounded theory is that it has been developed with co-researchers to not only understand the phenomenon, but to plan action for change.

### Limitations of this study

Limitations of this study include limited time of the lead researcher (MRM) in the field and its potential impact upon the two-way approach to research. This was mitigated to some degree due to previously established relationships with research partners while working on the large multi-site study, previous time spent living and/or working at the field sites and continued research with RT, a senior researcher from PNG who has since commenced her own PhD in the field of male circumcision for HIV prevention. The limited time at the sites during the research also impacted on the way the grounded theory method of theoretical sampling was enacted. Time at each field site was often approximately one week. Sampling challenges were encountered as time was limited, however these were somewhat overcome by the support of research partners who negotiated with co-researchers with particular characteristics to participate in the study (for example university faculty staff, married, single, health worker) prior to the researchers re-entering the field.

## Conclusions

Women in PNG know a lot about male circumcision and penile modification, despite it being ‘men’s business’. Women who have had opportunities for education have a greater range of choices and an increased opportunity to act upon these choices. However, women can only exercise their power of choice in the context of safety. This research has shown that women’s ambivalence previously reported about male circumcision occurs in a context of lack of safety, one expression of gender inequality in PNG. Women who feel safe are able to make choices and experience less ambivalence about male circumcision. The use of the transformational grounded theory methodology has provided a means to understand how women experience and manage male circumcision and penile modification in PNG, including HIV prevention and has identified opportunities for health promoting action.

## Abbreviations

HIV: Human immunodeficiency virus
PNG: Papua New Guinea
